# High Bleeding Risk Patients Undergoing Percutaneous Coronary Intervention: How to Choose the Optimal Antithrombotic Therapy, Its Duration and the Right Stent; A Comprehensive Review of the Current Literature and Proposed Treatment Algorithms

**DOI:** 10.3390/jcm15208035

**Published:** 2026-10-16

**Authors:** Charalampos Kakderis, Matthaios Didagelos, Thomas Zegkos, Konstantinos C. Theodoropoulos, Antonios Kouparanis, Georgios Samprokatsidis, Eleftheria Baltagianni, Stylianos Daios, Athinodoros Nikitopoulos, Thomas Gossios, Vasileios Kamperidis, George Kassimis, Stergios Tzikas, Antonios Ziakas

**Affiliations:** 1 1st Cardiology Department, AHEPA University Hospital, Aristotle University of Thessaloniki, 54636 Thessaloniki, Greece; 2 2nd Cardiology Department, Hippokration University Hospital, Aristotle University of Thessaloniki, 54636 Thessaloniki, Greece; 3 3rd Cardiology Department, Hippokration University Hospital, Aristotle University of Thessaloniki, 54636 Thessaloniki, Greece

**Keywords:** high-bleeding-risk patients, percutaneous coronary interventions, antithrombotic therapy, drug-eluting stents

## Abstract

High bleeding risk (HBR) patients represent a significant proportion of those who undergo percutaneous coronary interventions (PCI). The accurate diagnosis of the HBR population is essential because they suffer significantly higher rates of mortality and bleeding compared with non-HBR patients. Although many bleeding risk scores have been developed, the Academic Research Consortium for HBR (ARC–HBR) criteria have established their superiority over the others. In HBR patients without an indication for oral anticoagulant (OAC), shortening the duration of dual antiplatelet therapy (DAPT) is an effective way to minimize bleeding complications in HBR patients with chronic coronary syndromes (CCS). In cases of an acute coronary syndrome (ACS) however, HBR patients combine increased thrombotic burden with an augmented risk of bleeding. Early antiplatelet interruption could result in stent thrombosis with catastrophic consequences. Therefore, in HBR patients with an ACS choosing the optimal DAPT duration, the right P2Y12 inhibitor and the correct time for de-escalation from a potent P2Y12 inhibitor to clopidogrel are of paramount importance. Similar considerations apply to patients with an OAC indication who, according to the ARC–HBR criteria, are HBR. Although new-generation drug-eluting stents (DES) have been developed to induce faster stent endothelization, only few DES have been examined in a HBR population, and these stents should be preferred. The aim of this review is to present the current knowledge of the HBR population and provide comprehensive algorithms to optimally treat HBR patients who undergo PCI with or without an OAC indication and for both ACS and CCS.

## 1. Introduction

**Figure 1:**
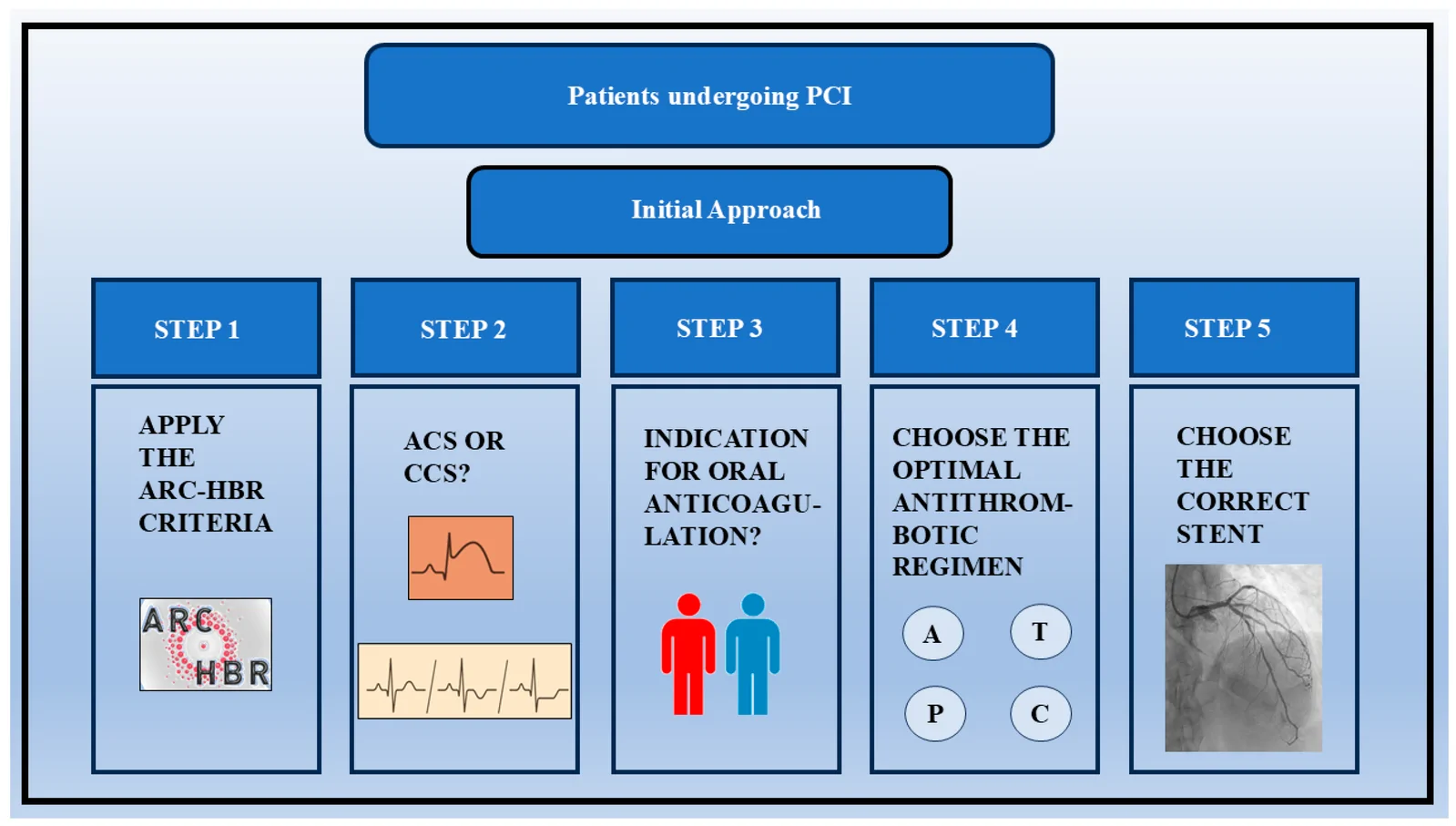
Initial stepwise approach of patients who undergo PCI in order to diagnose and treat HBR patients. A: aspirin; ACS: acute coronary syndrome; ARC–HBR: academic research consortium for high bleeding risk; C: clopidogrel; CCS: chronic coronary syndrome; HBR: high bleeding risk; P: prasugrel; PCI: percutaneous coronary intervention; T: ticagrelor.

Percutaneous coronary intervention (PCI) represents the gold-standard treatment and the default revascularization strategy for the majority of patients with acute coronary syndrome (ACS) [1]. In addition, PCI is indicated in patients with stable coronary artery disease (CAD) who remain symptomatic despite optimal medical treatment (OMT), as well as in certain groups of patients with chronic coronary syndrome (CCS) to improve their prognosis, such as those with functionally significant severe left main (LM) stenosis amenable to PCI [2]. After revascularization and stent implantation, patients receive dual antiplatelet therapy (DAPT), a combination of aspirin with a P2Y12 inhibitor, for the first period, followed by lifelong single antiplatelet therapy (SAPT) [2]. Severe bleeding can become a life-threating complication in patients after PCI, resulting in DAPT discontinuation and stent thrombosis (ST), particularly during the first days after the drug-eluting stent (DES)’s deployment. Therefore, it is of paramount importance to identify high bleeding risk (HBR) patients undergoing PCI [3]. The aim of our review was to conduct a comprehensive literature study in order to properly diagnose HBR patients who undergo PCI, identify their optimal antithrombotic regimen and its duration, present the stents which have proven their efficacy and safety in this population, and imply strategies to reduce their bleeding complications. This narrative literature review was conducted using the PubMed database. Relevant studies were identified using combinations of the following keywords: “High bleeding risk patients,” “Percutaneous coronary intervention,” and “Bleeding after percutaneous coronary intervention.” Articles published in English from inception to 2026 were considered. Additional relevant references were identified by screening the reference lists of the selected articles. The literature was reviewed and synthesized narratively according to the main themes of the topic. The initial stepwise approach of patients who undergo PCI in order to diagnose and treat HBR patients is analytically presented in Figure 1.

## 2. HBR Definition and Major Bleeding Risk Scores for Patients Undergoing PCI

The identification and management of HBR patients undergoing PCI are of paramount importance to reduce their bleeding complications and mortality rates. Consensus documents from the Academic Research Consortium for HBR (ARC–HBR) proposed that HBR patients undergoing PCI are defined as those with a risk of bleeding academic research consortium (BARC) [4] type 3 or 5, bleeding of ≥4%, or a risk of intracranial hemorrhage (ICH) of ≥1% at one year [5,6]. Although the HBR population was at first underrepresented or even excluded in randomized clinical trials (RCT), numerous contemporary bleeding risk scores have been developed to identify these patients.

### 2.1. The ARC–HBR Criteria

The ARC–HBR criteria represent the most pragmatic and valuable bleeding risk model [5]. The ARC–HBR classification consists of fourteen major and six minor criteria [6]. A major criterion is defined as any criterion that, in isolation, confers a risk of BARC 3 or 5 bleeding of ≥4% or a risk of ICH of ≥1% at one year. A minor criterion is defined as any criterion that, compared with its absence, confers an increased risk of BARC 3 or 5 bleeding of <4% at one year. Recently, a scientific expert panel was also created to propose the principles of design for trials in patients at high bleeding risk [7]. One major or two minor criteria can accurately identify HBR patients and have been comprehensively validated for bleeding outcomes in large cohorts. More specifically, in the Bern PCI Registry including 12,121 patients undergoing PCI [8], the ARC–HBR criteria were more sensitive in identifying patients who will suffer BARC 3 or 5 bleeding than the PRECISE DAPT [9] or the PARIS [10] bleeding risk scores, but with the cost of lower specificity [8]. All the ARC–HBR criteria are presented in detail in Table 1.

In addition, other registries, such as the multicenter CREDO–Kyoto registry, examined the efficacy of the ARC–HBR criteria in predicting bleeding complications by applying the ARC–HBR criteria in patients who underwent their first PCI [11]. At 5 years, the primary bleeding endpoint as well as the combined ischemic endpoint were significantly higher in the HBR group compared with the non-HBR group. Moreover, patients with one major ARC–HBR criterion, with the exception of cirrhosis, had a bleeding risk higher than ≥4% at 1 year. The same risk was applied in patients with one minor criterion, with the exception of prior ischemic stroke. The risk of bleeding was also augmented as the number of ARC–HBR criteria were increasing (≥3 majors: 49.9%, 2 majors: 30.6%, 1 major: 18.5%, ≥2 minors: 14.7%, and no-HBR: 6.6%, *p* < 0.0001) [11].

### 2.2. The PRECISE DAPT Score

The PRECISE DAPT score is a simple tool which consists of five variables (age, hemoglobin, creatinine clearance, white blood cell count, previous spontaneous bleeding) and was created to predict out-of-hospital bleeding complications in patients receiving DAPT after PCI [9,12]. A PRECISE-DAPT score of 25 or higher indicates HBR patients. Lim et al. [13] compared the performance of the ARC–HBR and the PRECISE DAPT bleeding scores in 7562 patients who were treated with DAPT after PCI. By applying the ARC–HBR criteria, 27% of them were HBR, whereas 38% were characterized as HBR with the PRECISE DAPT score. Overall, 22% had discordant HBR categorization between the two bleeding scores. Although both of them identified the patients at increased bleeding risk who were also at higher risk for ischemic complications, the PRECISE DAPT criteria underestimated the bleeding complications. The observed risks of bleeding were higher than those predicted by the PRESICE DAPT score across almost all score levels [13]. In addition, novel bleeding risk scores, such as PRECISE-HBR, offer significant improvements in predicting bleeding complications after PCI; however, further evaluation is required to assess their impact on everyday clinical practice [14].

### 2.3. The CRUSADE Score for In-Hospital Bleeding Complications

The CRUSADE bleeding score was developed to estimate the risk of in-hospital bleeding events in patients with non-ST-segment elevation myocardial infarction (NSTEMI) [15]. Although this score has also been used to accurately discriminate patients with ST-segment elevation myocardial infarction (STEMI) treated with PCI who are at risk of in-hospital major bleeding [16], the ARC–HBR criteria have demonstrated superior predictive ability for in-hospital bleeding compared with the CRUSADE score [17].

## 3. HBR Patients Undergoing PCI Without an Indication for Oral Anticoagulation (OAC)

**Figure 2:**
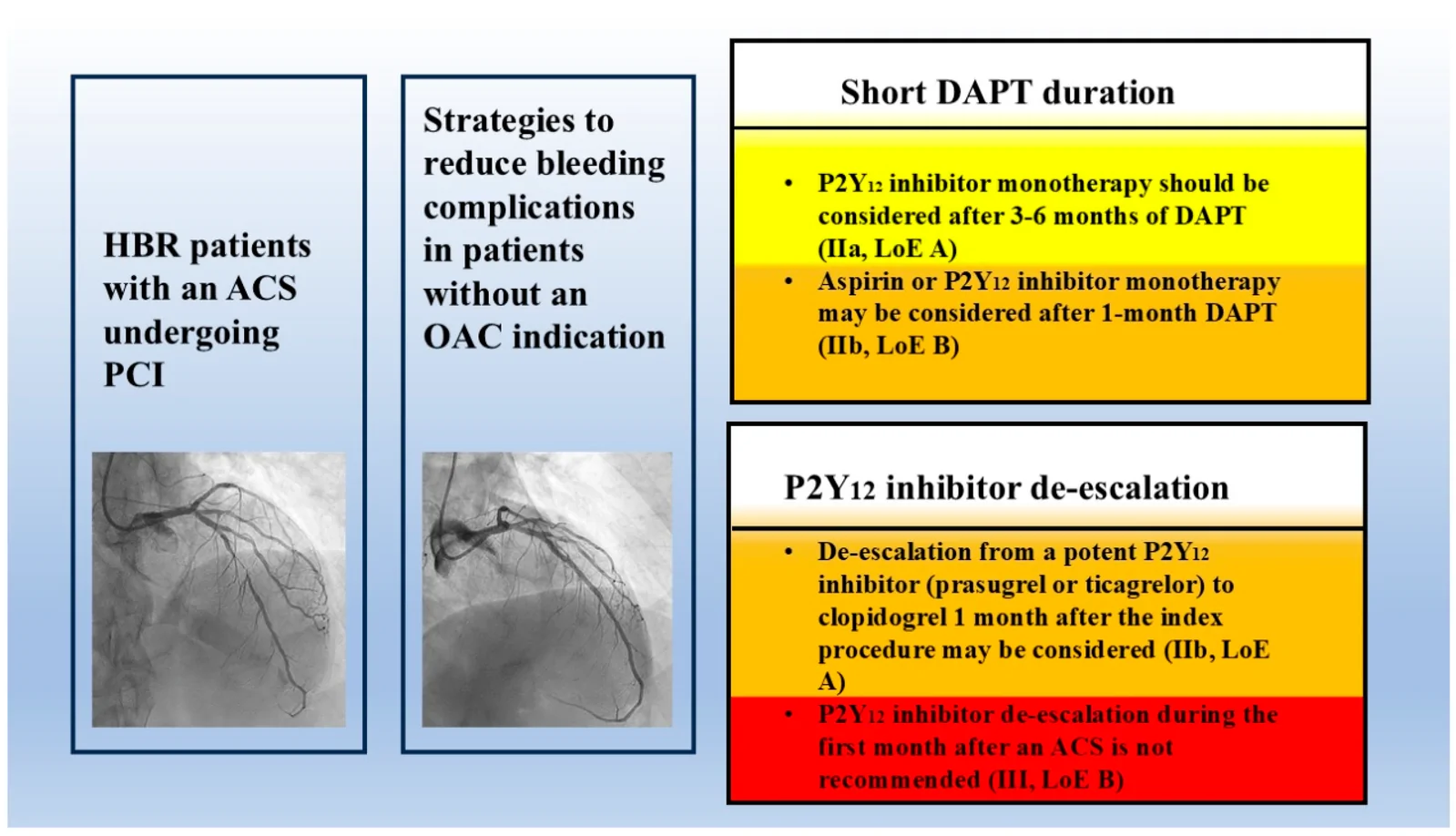
Antithrombotic strategies to reduce bleeding risk in HBR patients with an ACS who undergo PCI. Yellow color: Class IIa, should be considered, orange color: Class IIb, may be considered, red color: Class III, is not recommended. ACS: acute coronary syndrome; DAPT: dual antiplatelet therapy; HBR: high bleeding risk; LoE: level of evidence; OAC: oral anticoagulation.

The optimal DAPT duration after PCI in HBR patients remains one of the most challenging questions in contemporary clinical practice. Shortening the DAPT duration in these patients could minimize their bleeding complications without increasing the ischemic events, taking into consideration the concomitant ischemic risk and clinical presentation (ACS or stable CAD). Although many studies have examined the safety and efficacy of abbreviated DAPT combinations [18,19,20,21,22,23], very few have focused solely on HBR patients. Antithrombotic strategies aimed at reducing bleeding risk in HBR patients with ACS undergoing PCI are presented in Figure 2.

### 3.1. Ticagrelor Monotherapy After 3 Months of DAPT with Aspirin and Ticagrelor in HBR Patients

In the TWILIGHT-HBR trial [24], a prespecified analysis of the TWILIGHT trial [21], 1064 HBR patients who were treated with DES implantation received either ticagrelor with placebo or ticagrelor with aspirin after 3 months of DAPT with aspirin and ticagrelor and were monitored for 1 year. This trial included only patients who adhered to treatment and did not experience major bleeding or ischemic events during the first three months after PCI. Ticagrelor monotherapy was associated with a lower incidence of the primary endpoint of bleeding BARC 2, 3 and 5 at 1 year compared with the ticagrelor and aspirin combination, while no difference was observed in the secondary endpoints of death, myocardial infarction (MI), or stroke between the groups [24]. In another subgroup analysis of the TWILIGHT, the TWILIGHT-ACS, among 4614 patients with NSTEMI-ACS, ticagrelor monotherapy also reduced bleeding BARC 2, 3, and 5 by 53% compared with DAPT, while the rate of the composite thrombotic endpoint of death, MI and stroke also remained the same between the two groups [25].

In a sub-analysis from the TICO trial, Lee et al. [26] examined the impact of ticagrelor monotherapy after three months of DAPT compared with a 12-month DAPT regimen consisting of aspirin with ticagrelor in HBR patients with an ACS treated with DES. Patients who suffered adverse events during the first 3 months after PCI were excluded. In total, 453 patients (15.2%) were identified as HBR with the ARC–HBR criteria and 504 (16.9%) with the PRECISE DAPT score. Contrary to the TWILIGHT HBR study which did not include STEMI patients, in the TICO trial, 36% of the patients presented with STEMI [20]. The primary outcome was a net adverse clinical event (NACE) defined as the composite of major bleeding and major adverse cardiac and cerebrovascular events (MACCEs). The primary endpoint was higher in HBR group, applying both the ARC–HBR and the PRECISE DAPT criteria at one year. More importantly, ticagrelor monotherapy after 3 months of DAPT in the HBR group was associated with lower primary outcome compared with the 12-month DAPT group [26] with similar therapy effect for HBR and non-HBR patients. These outcomes were consistent regardless of HBR classification by ARC–HBR or PRECISE DAPT criteria.

Other trials, not exclusively focusing on HBR population, have also examined ticagrelor monotherapy after a short initial period of DAPT in patients with ACS confirming the increased safety and similar efficacy of ticagrelor monotherapy after short term DAPT rendering this strategy as a reasonable approach for HBR patients [27,28,29]. To lend more support, recent guidelines of the American College of Cardiology/American Heart Association (ACC/AHA) for the management of patients with ACS propose the ticagrelor monotherapy after ≥1 month of DAPT with ticagrelor in order to reduce bleeding risk [30].

### 3.2. One-Month DAPT and SAPT (With Aspirin or P2Y12 Inhibitor) Afterwards

In a post hoc subgroup analysis of the STOPDAPT-2 trial, Watanabe et al. [31] examined the effect of 1-month DAPT (consisting of aspirin with clopidogrel) followed by clopidogrel alone in HBR patients. Patients who underwent PCI with implantation of a Xience DES and had no major adverse in-hospital events were considered eligible for inclusion in the study. The abbreviated regimen was compared with 12-month DAPT (aspirin plus clopidogrel), and all patients were monitored for 12 months. The primary endpoint was a 1-year composite of cardiovascular and bleeding outcomes. The major secondary cardiovascular endpoint was a composite of cardiovascular death (CVD), MI, definite ST, and ischemic or hemorrhagic stroke, and the major secondary bleeding endpoint was the bleeding defined as thrombolysis in myocardial infarction (TIMI), major or minor. In this sub-analysis, from the total 1054 HBR patients, only 139 (13.2%) presented with STEMI, 41 (3.9%) with NSTEMI, and 130 (12.3%) with unstable angina (UA), while the majority of the participants had stable CAD (744 patients, 70.6%). At 12 months, the primary endpoint was not different between the two groups, as well as the major secondary cardiovascular endpoint. On the other hand, the major secondary bleeding endpoint was markedly lower in the abbreviated DAPT group compared with the default strategy.

The MASTERDAPT trial was the landmark trial for the management of the HBR patients [32]. More specifically, 4579 HBR patients treated with PCI received DAPT for 1 month and then were randomized to continue with SAPT (abbreviated treatment) or DAPT for at least 5 more months (standard treatment). Eligible patients were required to remain free of adverse cardiovascular events during the first month following PCI. Patients with an OAC indication after receiving 1-month DAPT were randomized also to receive the abbreviated regimen (SAPT and OAC for up to 6 months, and OAC monotherapy thereafter) or the standard therapy (at least 2 more months DAPT with OAC and continue SAPT with OAC until 12 months after the procedure) [33]. In the MASTERDAPT trial, the majority of the participants had stable CAD and only 538 patients (11.7%) presented with STEMI, and 1153 (25%) with NSTEMI. In one year, one-month DAPT was non-inferior compared with the conventional regimen in the primary NACE endpoint. NACE and MACCE were similar between the abbreviated and the default therapy, both in patients with an OAC (*n* = 1666) and without an OAC indication (*n* = 2913) [33]. On the other hand, bleeding BARC 2, 3, or 5 did not significantly differ in patients with OAC indication (HR, 0.83 [95% CI, 0.62–1.12]), but was lower with abbreviated antiplatelet therapy (APT) in patients without OAC indication (HR, 0.55 [95% CI, 0.41–0.74]) [32,33]. At 15 months after PCI, the benefits of the abbreviated DAPT therapy were maintained [34]. In contrast to the MASTERDAPT trial, in the HOST-BR study, 1-month DAPT after PCI in HBR patients did not meet the non-inferiority criterion compared with 3-month DAPT for NACE (all-cause death, MI, ST, stroke, or major bleeding) at 12 months. Overall, 1598 HBR patients received DAPT for 1 or 3 months, and 482 (30%) presented with MI. However, this study included only East Asian patients [35].

A recent meta-analysis of major RCTs also confirmed the benefits of the abbreviated DAPT strategy in HBR patients [36]. The abbreviated DAPT (1–3 months) resulted in a reduction in major or clinically relevant non-major (CRNM) bleeding, major bleeding and CVD compared with standard DAPT (≥6 months). In addition, no difference was observed in all-cause death, major adverse cardiovascular events (MACE), MI, or ST. These findings remained consistent regardless of the HBR definition or clinical presentation (ACS or CCS) [36]. Shortening DAPT duration is safe even in HBR patients with concomitant high ischemic risk. In the OPT-BIRISK trial, in patients with ACS who were at high ischemic and high bleeding risk (birisk) undergoing PCI, switching to clopidogrel monotherapy after 9–12 months of DAPT and continuing it for an additional 9 months resulted in less bleeding than continued DAPT with aspirin plus clopidogrel, without increasing the risk of ischemic events [37].

### 3.3. Omitting Aspirin Immediately and During the First Month After PCI in HBR Patients Is Not Recommended

In the subgroup analysis of the STOPDAPT-3 trial [38], 3258 HBR patients were analyzed [39]. In total, 1803 patients (55.3%) presented with an ACS and 1455 (44.7%) with a CCS. In the non-aspirin group, prasugrel monotherapy (3.75 mg/day) was continued up to 1 month after PCI and was replaced with clopidogrel up to 12 months. Patients in the DAPT group (aspirin with prasugrel) discontinued prasugrel at 1 month and received aspirin up to 1 year. At 1 month, the co-primary bleeding endpoint was similar between the two groups, both in the ACS and in the non-ACS HBR patients. The primary cardiovascular endpoint was numerically higher in the prasugrel monotherapy group only in ACS patients compared with the DAPT group (*p* = 0.07), while the two regimens did not differ in the non-ACS patients. Consistent with the results of the STOPDAPT-3 trial, the recently presented PREMIUM trial demonstrated that prasugrel monotherapy in patients with STEMI treated with primary PCI was not non-inferior to the DAPT control group (aspirin with prasugrel) regarding the primary composite outcome of all-cause death, MI or stroke at 12 months [40]. In conclusion, these results indicate that completely omitting aspirin in HBR patients undergoing PCI is not generally safe, does not reduce bleeding, and should therefore be avoided.

### 3.4. P2Y12 Inhibitor De-Escalation to Reduce Bleeding Risk

The first period after an ACS is characterized by excessive thrombotic burden. Although the more potent DAPT combinations (aspirin with prasugrel or ticagrelor) reduce the ischemic complications, prolonged administration of these P2Y12 inhibitors can lead to bleeding adverse events. Therefore, de-escalation from these P2Y12 inhibitors to one less potent (clopidogrel) after the initial period of an ACS has been established as an alternative regimen for HBR patients [41,42,43,44,45].

In the TALOS-AMI HBR substudy, 589 HBR patients (22.4%) of the total 2625 were analyzed [46]. One month after PCI, patients without any adverse clinical events were randomized to de-escalate the P2Y12 inhibitor (from ticagrelor to clopidogrel) or to continue aspirin with ticagrelor. The primary endpoint rate of NACE (a composite of CVD, MI, stroke or bleeding BARC 2, 3 and 5) as well as major bleeding BARC 3 or 5 were less common in the de-escalation group compared with the control group [46].

Laudani et al. [47] conducted a meta-analysis to compare a short duration of DAPT with de-escalation. This meta-analysis of 29 studies included, in total, 50,602 patients who underwent PCI for an ACS. The primary endpoint was all-cause mortality, while NACE, MACE and their components were the secondary outcomes. Although the two strategies reported similar rates of all-cause death, short DAPT duration resulted in a reduced risk of major bleeding compared with de-escalation [47].

**Figure 3:**
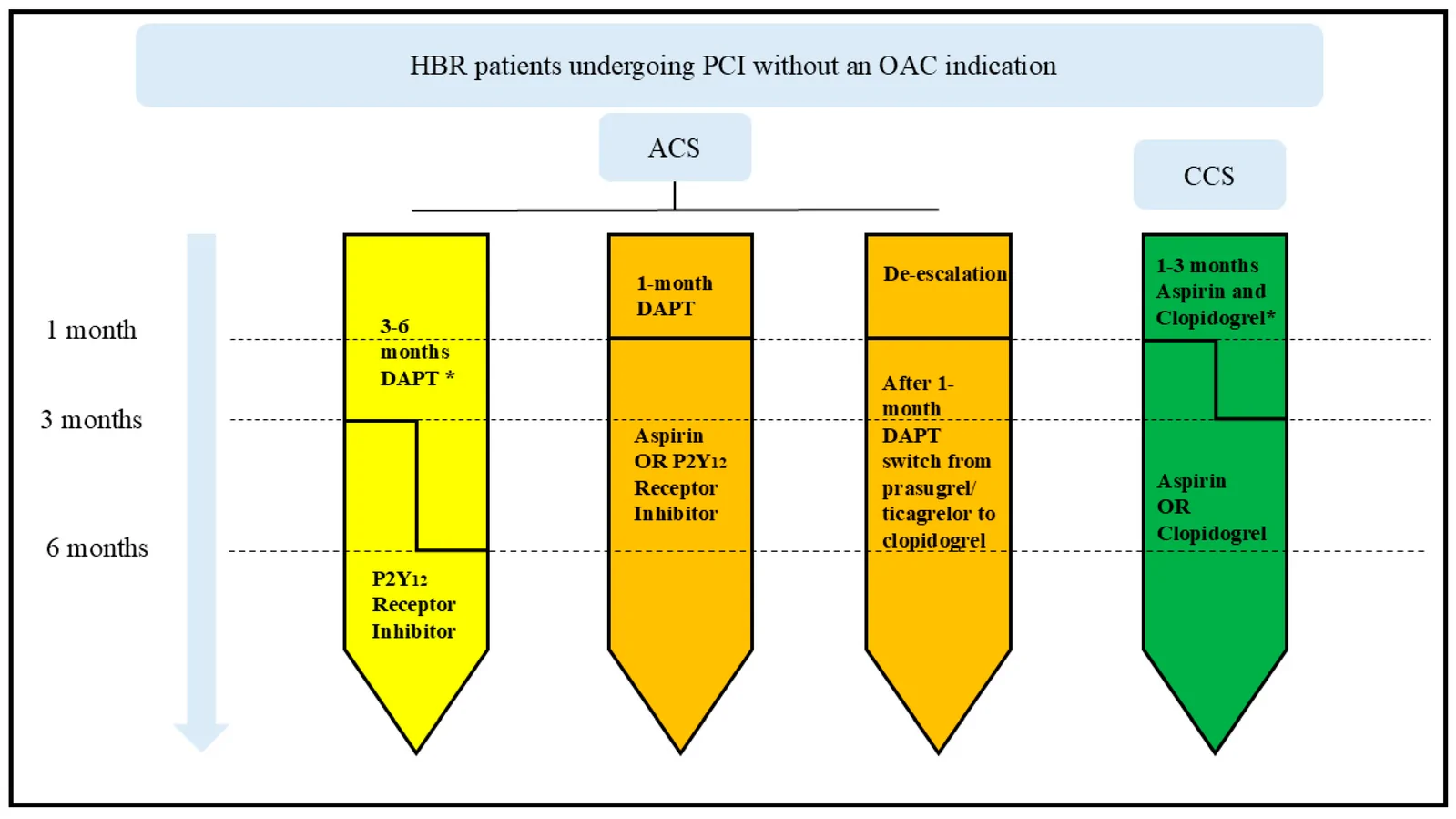
High-bleeding-risk patients without an OAC indication undergoing PCI for an ACS or CCS. Green color: Class I, is recommended or is indicated; yellow color: Class IIa, should be considered; orange color: Class IIb, may be considered. * Patients not at high ischemic risk. High ischemic risk patients include: complex CAD and at least one criterion; technical aspects: at least 3 stents implanted, at least 3 lesions treated, total stent length > 60 mm, history of stent thrombosis on antiplatelet treatment, complex revascularization (left main, chronic total occlusion, stenting of last patent vessel, bifurcation stenting with ≥2 stents implanted); risk enhancers: diabetes mellitus, recurrent MI, multivessel CAD, premature (<45 years) or accelerated (new lesion within a 2-year timeframe) CAD, concomitant systemic inflammatory disease, polyvascular disease (CAD plus PAD), CKD with eGFR 15–59 mL/min/1.73 m^2^. ACS: acute coronary syndrome; CAD: coronary artery disease; CCS: chronic coronary syndrome; CKD: chronic kidney disease; DAPT: dual antiplatelet therapy; eGFR: estimated glomerular filtration rate; HBR: high bleeding risk; MI: myocardial infarction; OAC: oral anticoagulant; PAD: peripheral arterial disease; PCI: percutaneous coronary intervention.

Summarizing all these results, it is clear that HBR patients with an ACS represent one of the most challenging groups to choose the optimal DAPT duration. High-risk patients like those with STEMI were excluded or underrepresented in the majority of these trials. According to the European Society of Cardiology (ESC) guidelines for ACS, for HBR patients who are event-free after the implementation of 3 to 6 months of DAPT, SAPT monotherapy (preferably with a P2Y12 inhibitor) should be considered as the most appropriate regimen when patients are not at high ischemic risk [Class of recommendation IIa, Level of Evidence (LoE) A] [1]. One-month DAPT may be considered only when the bleeding risk significantly surpasses the thrombotic risk (Class of recommendation IIb, LoE B). In addition, de-escalation of a potent P2Y12 inhibitor (ticagrelor or prasugrel) to clopidogrel may be considered to reduce bleeding complications (Class of recommendation IIb, LoE A). On the other hand, it is well established that HBR patients with CCS who undergo PCI can discontinue DAPT after 1 to 3 months and continue with SAPT, especially when they are not at high ischemic risk (Class of recommendation I, LoE A). All these results are summarized in Figure 3 and the major trials are presented analytically in Table 2.

## 4. HBR Patients with an OAC Indication Undergoing PCI

### 4.1. Abbreviating Triple Antithrombotic Treatment

Chronic OAC treatment is essential and necessary in patients with mechanical valves as well as in the majority of patients with atrial fibrillation (AF). OAC treatment is a major criterion according to ARC–HBR criteria; therefore, patients with an OAC indication who undergo PCI are considered HBR. Triple antithrombotic therapy (TAT) consisting of DAPT with an OAC after PCI is excessively augmenting bleeding complications while the risk of early stent thrombosis is always a pivotal consideration when early antiplatelet interruption occurs. Therefore, the balance between the thrombotic and bleeding complications in patients who require OAC treatment has been extensively examined in many RCTs. It should be noted, however, that the studies that established the role of direct oral anticoagulants (DOACs) in the treatment of patients with AF undergoing PCI excluded patients with mechanical heart valves, who should be treated exclusively with vitamin K antagonists (VKAs). The WOEST study was one of the first trials which evaluated single antiplatelet use with an OAC after coronary revascularization [48]. More specifically, 573 patients requiring OAC received either dual antithrombotic therapy (DAT) consisting of clopidogrel with an OAC or TAT consisting of aspirin, clopidogrel and OAC after PCI. In case of DES implantation or ACS, clopidogrel was maintained for 12 months. Any bleeding and the combined ischemic endpoint [death, MI, stroke, target vessel revascularization (TVR), ST] were lower in the DAT group, while major bleeding were similar between the two groups. Abbreviated TAT has also been examined in the ISAR-TRIPLE trial [49]. In total, 614 patients with an OAC indication were randomized to receive clopidogrel for either 6 weeks or 6 months in addition to aspirin and a vitamin K antagonist (VKA) after DES implantation. At 9 months, the primary cumulative outcome of death, MI, stroke, ST or TIMI major bleeding was similar between the two groups, as well as the secondary ischemic and bleeding endpoints.

A recent meta-analysis, which included 10,234 patients with AF who underwent PCI and compared DAT with TAT, noted that although major or CRNM bleeding was markedly lower in the DAT group (*p* < 0.0001), ST occurred more frequently in the DAT group (*p* = 0.04). In addition, a trend towards a higher risk of MI was also noted. These results highlight the paramount importance of aspirin in the first period after stent implantation even for a short period of time [50].

### 4.2. Direct Oral Anticoagulants Are Preferred to VKA

In patients with an ACS or CCS undergoing PCI who require OAC, DOACs are preferred to VKA, unless contraindicated [51,52]. In the PIONEER AF-PCI trial, 2124 patients with nonvalvular AF who were treated with DES implantation were randomized in a 1:1:1 ratio to receive 15 mg rivaroxaban plus a P2Y12 inhibitor for 12 months (group 1), 2.5 mg rivaroxaban twice daily plus DAPT for 1, 6 or 12 months (group 2) or standard therapy with VKA plus DAPT for 1, 6, or 12 months (group 3) [53]. At 12 months, TIMI major and minor bleeding as well as bleeding requiring medical attention occurred less frequently in the rivaroxaban groups compared with the VKA group, while the ischemic outcomes were similar between the three groups. In this trial, 639 patients (30%) presented with an MI and 2004 patients received clopidogrel (94%) as the P2Y12 inhibitor.

In the AUGUSTUS trial, 4614 patients with AF who had ACS or underwent PCI and were planned to receive a P2Y12 inhibitor were analyzed [51]. These patients were randomized in a factorial two-by-two design to receive apixaban or VKA and to receive aspirin or placebo for 6 months. The primary endpoint of major or CRNM bleeding occurred in 10.5% of the patients in the apixaban group compared with 14.7% in the VKA group (*p* < 0.001 for both non-inferiority and superiority), and in 16.1% of the patients receiving aspirin compared with 9% of those receiving placebo (*p* < 0.001). In addition, patients in the apixaban group also had lower rates of death or hospitalization compared with the VKA group, and similar rates of ischemic events. In the AUGUSTUS trial, 1714 patients (37%) presented with an ACS and were treated with PCI, and clopidogrel was the preferable P2Y12 inhibitor (4165 patients, 90%). An initial TAT for a short period after PCI is also supported from a meta-analysis of the DOAC studies [54].

In the REDUAL PCI trial, 2725 patients with AF who had undergone PCI received TAT consisting of warfarin, a P2Y12 inhibitor (clopidogrel or ticagrelor) and aspirin (for 1–3 months), or DAT consisting of dabigatran 110 mg and a P2Y12 inhibitor (clopidogrel or ticagrelor), or dabigatran 150 mg and a P2Y12 inhibitor [55]. Major or CRNM bleeding was substantially lower in the 110 mg DAT group compared with TAT. Moreover, dual therapy with dabigatran (combined) was non-inferior to TAT with respect to the rate of thromboembolic events. In the REDUAL PCI trial, 50.4% of the patients presented with an ACS. In the ENTRUST-AF PCI trial, 1506 patients with AF were randomized to receive edoxaban and a P2Y12 inhibitor for 12 months, or a VKA in combination with a P2Y12 inhibitor for 12 months and aspirin for 1–12 months after PCI [56]. At 1 year, major or CRNM bleeding complications occurred in 128 (17%) of 751 patients in the edoxaban group, and in 152 (20%) of 755 patients in the VKA group (*p* = 0.0010 for non-inferiority; *p* = 0.1154 for superiority), with similar ischemic events between the two groups. In total, 777 patients (52%) presented with an ACS, and clopidogrel was the most common P2Y12 inhibitor used. In addition, potent antiplatelet agents such as prasugrel or ticagrelor should not be used in combination with DOACs after PCI. In the EPIDAURUS trial, patients with AF and ACS undergoing PCI received either a DOAC with prasugrel or ticagrelor for 1 month, followed by clopidogrel thereafter, or a combination of a DOAC plus clopidogrel and in-hospital aspirin. The study was prematurely terminated because treatment with a potent P2Y12 inhibitor resulted in higher rates of bleeding without reducing the risk of ischemic complications [57]. Furthermore, contemporary trials support a regimen in which the P2Y12 inhibitor is interrupted after one year of PCI and the OAC monotherapy is continued [58]. It should be noted that all of these studies have several limitations. The study designs primarily focused on the safety of the antithrombotic therapy, while its efficacy could not be adequately assessed because of the small sample sizes.

**Figure 4:**
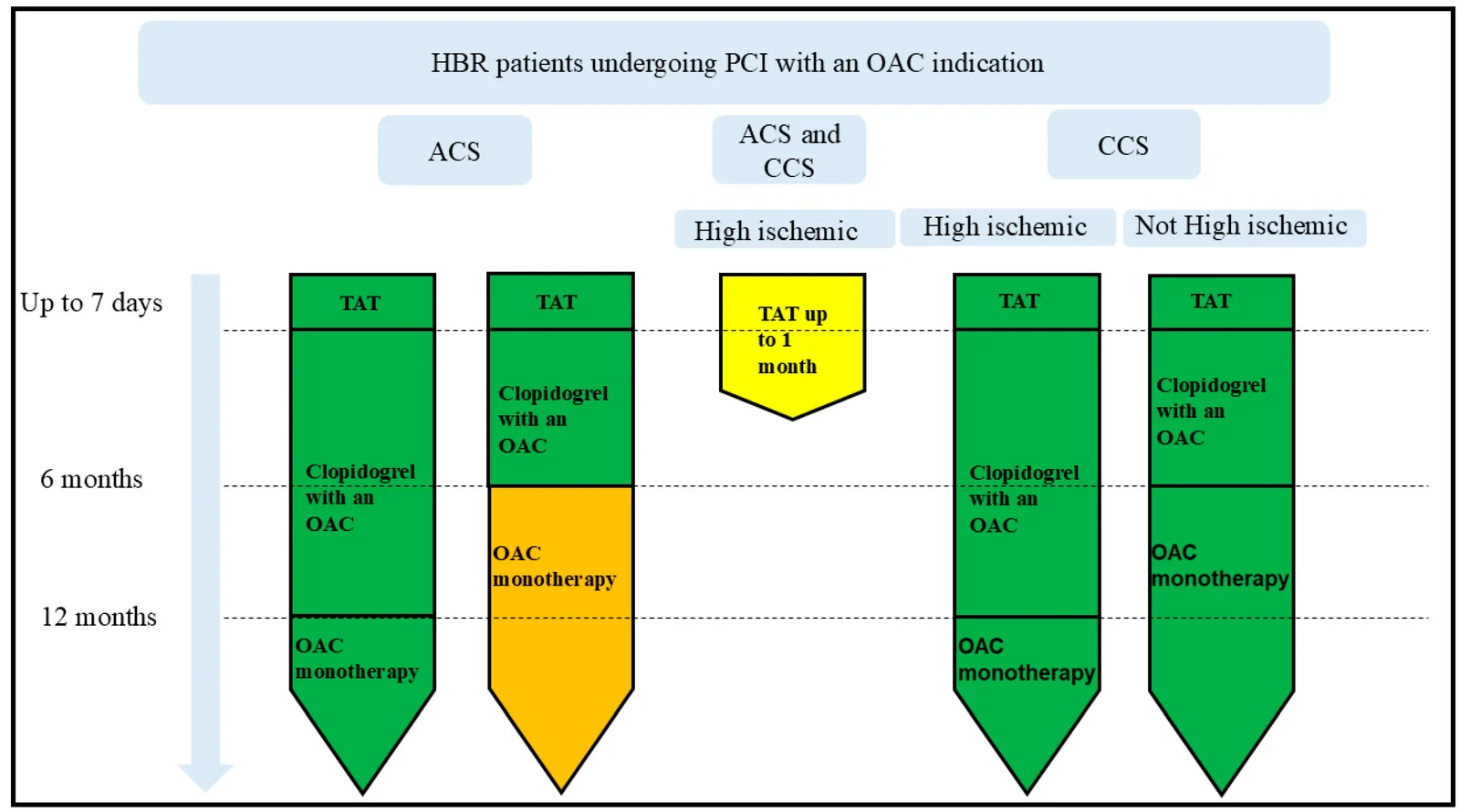
High-bleeding-risk patients with an OAC indication undergoing PCI for an ACS or CCS. Green color: Class I, is recommended or is indicated; yellow color: Class IIa, should be considered; orange color: Class IIb, may be considered. High ischemic risk patients include: complex CAD and at least one criterion: technical aspects: at least 3 stents implanted, at least 3 lesions treated, total stent length >60 mm, history of stent thrombosis on antiplatelet treatment, complex revascularization (left main, chronic total occlusion, stenting of last patent vessel, bifurcation stenting with ≥2 stents implanted); risk enhancers: diabetes mellitus, recurrent MI, multivessel CAD, premature (<45 years) or accelerated (new lesion within a 2-year timeframe) CAD, concomitant systemic inflammatory disease, polyvascular disease (CAD plus PAD), CKD with eGFR 15–59 mL/min/1.73 m^2^. ACS: acute coronary syndrome; CAD: coronary artery disease; CCS: chronic coronary syndrome; CKD: chronic kidney disease; eGFR: estimated glomerular filtration rate; HBR: high bleeding risk; MI: myocardial infarction; OAC: oral anticoagulant; PAD: peripheral arterial disease; PCI: percutaneous coronary intervention; TAT: triple antithrombotic therapy.

Summarizing all these results, it is clear that, in HBR patients with an OAC indication, a short period of TAT (less than one month) with aspirin administration reduces the rate of early ST and is recommended following PCI. After TAT, in ACS patients, it is recommended to continue with DAT consisting of P2Y12 inhibitor (clopidogrel) and a DOAC until 12 months, and with an OAC monotherapy afterwards. The European Society of Cardiology for the treatment of ACS suggests that discontinuation of DAT at 6 months may be considered (Class of recommendation IIb) with LoE B [1]. It should be highlighted that, while rivaroxaban was used at a dose of 15 mg once daily in the PIONEER AF-PCI trial (or 10 mg once daily in patients with a creatinine clearance of 30–50 mL/min), DOACs are recommended at the dose indicated for stroke prevention when used as part of the combination antithrombotic therapy (Class of recommendation I, LoE A). However, when concerns about bleeding outweigh the risk of ischemic stroke, patients receiving dabigatran or rivaroxaban should be considered for treatment with lower doses of 110 mg (twice daily) or 15 mg (once daily), respectively, in preference to dabigatran 150 mg or rivaroxaban 20 mg, in order to minimize the risk of bleeding (Class of recommendation IIa, LoE B). When the thrombotic burden surpasses the bleeding risk, continuation of TAT for 1 month after PCI should be considered (Class of recommendation IIa, LoE B) in both ACS and CCS procedures. In patients with a CCS undergoing revascularization, the default strategy is TAT for up to seven days, DAT consisting of clopidogrel and DOAC for 6 months in patients not at high ischemic risk and for 12 months in patients at high ischemic risk (Class of recommendation I, LoE A). One year after the procedure, continuation only with an OAC is recommended. All of these results are summarized in Figure 4 and the major trials are presented in Table 3.

## 5. Stents with Thin and Ultra-Thin Struts for HBR Patients

### 5.1. Bare-Metal Stents or DES for HBR Patients?

First-generation stents were noted to have increased late thrombosis rates and prolonged DAPT was used to minimize this complication [59]. For this reason, many HBR patients were initially treated with bare-metal stents (BMS) in order to shorten DAPT duration. The LEADERS FREE trial was one of the first major studies to compare a umirolimus-coated stent, also known as biolimus A9, with BMS in HBR patients who underwent PCI [60]. In this study, 2466 HBR patients were randomized to receive, in a 1:1 ratio, one of these two stents and continue DAPT for 1 month. At 390 days, the primary safety endpoint of cardiac death (CD), MI or ST, was substantially lower in the DES group [60]. In addition, the primary efficacy endpoint of target lesion revascularization (TLR) was also lower in the DES group. The LEADERS FREE trial revolutionized the invasive treatment of HBR patients and established the use of DES over BMS in this population.

Age represents one minor criterion according to the ARC–HBR classification, and it is well established that elderly patients suffer more frequent major bleeding complications [61]. After the LEADERS FREE trial, the SENIOR trial investigated the efficacy and safety of DES compared with BMS in elderly patients (≥75 years old) treated with a short duration of DAPT [62]. Patients treated with PCI received either a DES or a BMS and all of them were treated with one month of DAPT in the case of CCS, or with six-month DAPT in the case of an ACS. At one year, the composite primary endpoint of all-cause mortality, MI, stroke, or ischemia-driven TLR was markedly lower in the DES group compared with the BMS group (*p* = 0.02), while the two groups did not differ in bleeding complications and ST.

Valgimigli et al. [63] also conducted the ZEUS study, a multinational RCT, to compare zotarolimus-eluting stents (ZES) with BMS in HBR patients treated with PCI. At 1 year, the primary outcome of major adverse cardiovascular events (MACE) was statistically lower in the ZES group compared with BMS group. Moreover, definite or probable ST was also significantly reduced with the ZES stent [63]. The ZEUS trial was one of the first studies which proved the superiority of the new-generation DES compared with BMS in HBR patients. A recent meta-analysis has also confirmed these results [64].

### 5.2. New-Generation DES for HBR Population

In order to further reduce DAPT duration and bleeding complications, new-generation DESs with thin and ultra-thin struts have been developed to induce faster stent endothelization. According to the results of the post hoc analysis of the HOST-IDEA trial, even in patients undergoing complex PCI with a third-generation stent, 3–6 months of DAPT is associated with a reduced risk of bleeding without an increased risk of ischemic events compared with 12 months of DAPT [65]. In the MASTERDAPT trial, a biodegradable-polymer sirolimus-eluting stent, the Ultimaster (Terumo) DES, was used in the PCI procedures [32]. After the Ultimaster implantation, one-month DAPT resulted in same NACE and MACCE with less bleeding complications compared with the standard therapy (at-least-6-month DAPT) [32]. The efficacy and safety of the Ultimaster stent in HBR patients treated with short DAPT duration has also been confirmed in real-world registries [66].

The XIENCE Short DAPT program was a prospective, multicenter study to assess the use of Xience DES (Abbott), a cobalt–chromium everolimus stent, with concomitant short durations of DAPT in HBR patients with CCS and ACS treated with PCI [67,68]. This program enrolled, in total, 3652 HBR patients (1605 received DAPT for one month and 2047 for 3 months). The combined endpoint of all-cause mortality or MI was similar between the 3-month DAPT group and the 12-month DAPT historical control group, as well as between the 1-month DAPT group and the 6-month DAPT historical control group. Major bleeding BARC 3–5 was lower in both the abbreviated DAPT groups compared with their historical controls. It should be noted that the XIENCE Short DAPT program excluded patients presented with STEMI and included only patients with CCS, UA or NSTEMI. Although the XIENCE Short DAPT program compared the Xience DES with historical control groups, Xience stents have been extensively studied in HBR populations, including in the STOPDAPT-2 and STOPDAPT-3 randomized controlled trials (RCTs) [31,39]. In conclusion, the Xience DES has been established as one of the most suitable stents for coronary revascularization in HBR patients [69].

The Resolute Onyx stent has also been extensively examined in HBR patients [70]. Windecker et al. [71] conducted the Onyx ONE Global study and compared the Resolute Onyx stent with the BioFreedom stent in a HBR population. A total of 1996 HBR patients with an indication for PCI (1003 in the Resolute Onyx stent group and 993 in the BioFreedom stent group) were treated with DAPT for one month after PCI and then continued with SAPT. The BioFreedom stents had already been used in the HBR population in the LEADERS FREE trial [60]. At 1 year, the Resolute Onyx stent was non-inferior to the BioFreedom stent in the composite primary outcome of CD, MI, and ST and in the secondary outcome of target lesion failure (the composite endpoint of CD, target-vessel MI or TLR) [71]. These results were also maintained two years after the PCI [72]. The Resolute Onyx stent has also proven its efficacy, even in HBR patients with diabetes mellitus (DM) [73].

Pivato et al. [74] conducted a multicenter study to access the use of Synergy bioresorbable polymer everolimus-eluting stent in a HBR population treated with PCI. A total of 443 HBR patients received DAPT for 1 month followed by SAPT and were monitored for 1 year. HBR patients who received the Synergy stent had a low incidence of the primary cumulative outcome of CD, MI or ST (4.82%), which was not inferior compared with the prespecified objective performance criterion of 9.4% [74]. In addition, in the EVOLVE Short DAPT study, 2009 HBR patients were enrolled and received DAPT for 3 months after implantation of the Synergy stent [75,76]. After DAPT discontinuation, patients received aspirin and were compared with a historical control group. In the Synergy group, the rate of MI or death was 5.6% compared with 5.7% of the prolonged DAPT historical control patients (*p* for non-inferiority = 0.0016). Therefore, it should be noted that the Synergy DES has only been evaluated in single-arm studies and studies with historical control groups, which do not allow for the establishment of comparative superiority.

**Figure 5:**
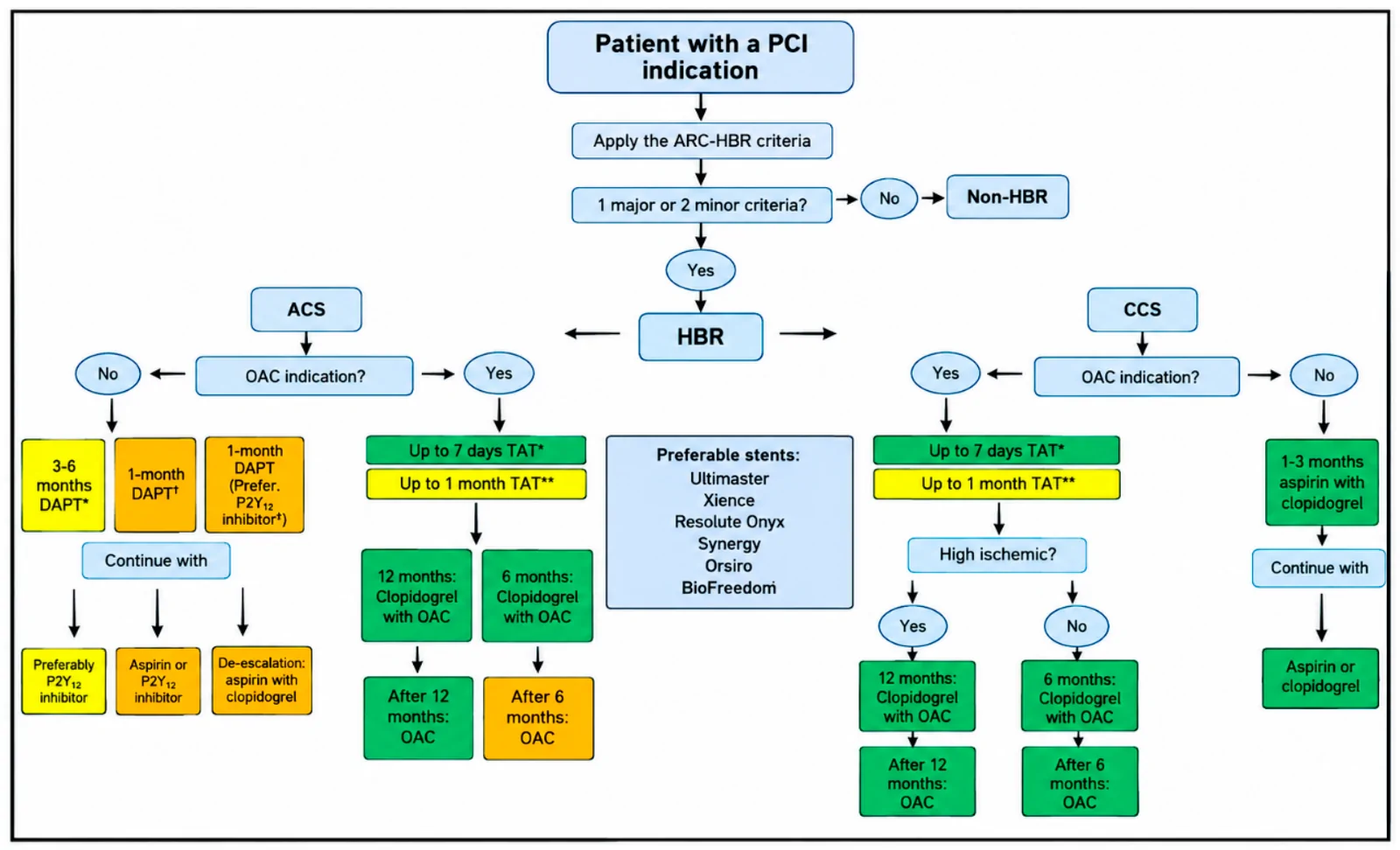
Proposed treatment algorithm to manage HBR patients who undergo PCI. Green color: Class I, is recommended or is indicated; yellow color: Class IIa, should be considered; orange color: Class IIb, may be considered. * Patients not at high ischemic risk, ** patients at high ischemic risk. †: Level of evidence: B, ‡: potent P2Y12 inhibitor. High ischemic risk patients include: complex CAD and at least one criterion: technical aspects: at least 3 stents implanted, at least 3 lesions treated, total stent length >60 mm, history of stent thrombosis on antiplatelet treatment, complex revascularization (left main, chronic total occlusion, stenting of last patent vessel, bifurcation stenting with ≥2 stents implanted); risk enhancers: diabetes mellitus, recurrent MI, multivessel CAD, premature (<45 years) or accelerated (new lesion within a 2-year timeframe) CAD, concomitant systemic inflammatory disease, polyvascular disease (CAD plus PAD), CKD with eGFR 15–59 mL/min/1.73 m^2^. ACS: acute coronary syndrome; ARC–HBR: Academic research consortium for high bleeding risk; CAD: coronary artery disease; CCS: chronic coronary syndrome; CKD: chronic kidney disease; DAPT: dual antiplatelet therapy; eGFR: estimated glomerular filtration rate; HBR: high bleeding risk; MI: myocardial infarction; OAC: oral anticoagulant; PAD: peripheral arterial disease; PCI: percutaneous coronary intervention; TAT: triple antithrombotic therapy.

The Orsiro DES, a biodegradable-polymer sirolimus-eluting stent, has also been established as a safe option for the HBR patients. In the Bioflow-DAPT study, Valgimigli et al. [77] enrolled HBR patients with a PCI indication to receive the Orsiro Mission (Biotronik) stent or the Resolute Onyx (Medtronic) stent. In total, 1948 patients were treated with DAPT for 1 month and then continued with SAPT. At 1 year, the primary cumulative outcome of CD, MI, or ST was similar between the two groups. Furthermore, a new stent, the Supraflex Cruz DES, has presented encouraging results in HBR populations [78,79]. Table 4 summarizes the main results of the stents which should be used in HBR patients. In addition, Figure 5 presents the proposed treatment algorithm to manage HBR patients who undergo PCI.

## 6. Other Strategies to Reduce Bleeding Complications

The radial artery is the default access site for coronary interventions in both HBR and non-HBR patients. Femoral access, compared with radial access, is associated with a higher risk of severe complications, such as major bleeding [80], and should generally be avoided in HBR patients when feasible. In addition, it is well established that in-hospital bleeding is associated with adverse long-term outcomes in patients with ACS [81]. The transradial access also reduces mortality rates, particularly in patients with moderate or severe anemia (baseline hemoglobin level < 11.0 g/dL), who, according to the ARC–HBR criteria, are considered to be at high bleeding risk (HBR) [82]. Moreover, anticoagulant doses have to be adjusted according to body weight and renal function, especially in elderly patients. Proton pump inhibitors should be used in patients receiving DAPT or OAC who are at high risk of bleeding, such as those with a history of gastrointestinal ulcer or hemorrhage. Moreover, in the OPTION trial, although the combination of indobufen and clopidogrel was associated with less bleeding than aspirin plus clopidogrel DAPT in patients with multivessel coronary artery disease undergoing PCI, patients with myocardial infarction were excluded from the study [83]. Finally, drug-coated balloons (DCBs) have shown encouraging results in the HBR population with stable CAD [84], although further studies are needed, particularly in HBR patients with ACS or those deemed to be at high ischemic risk.

## 7. Conclusions and Future Perspectives

Many contemporary studies like the TICO trial [20], the TWILIGHT trial [21], the T-PASS trial [27], the ULTIMATE-DAPT trial [28], as well as the prespecified analysis of the GLOBAL LEADERS trial [29], examined ticagrelor monotherapy after 1–3 months of DAPT (aspirin with ticagrelor) in patients with an ACS. Although these studies did not exclusively include HBR patients, ticagrelor monotherapy resulted in fewer bleeding compared with the prolonged DAPT regimen. In addition, the ischemic complications were similar between the two groups. Therefore, ticagrelor monotherapy following 1–3 months of DAPT represents an innovative treatment for patients at high bleeding risk. Furthermore, it should be noted that although DOACs have been established as the preferred anticoagulant therapy compared with VKAs, the optimal duration of DAT (clopidogrel with an DOAC) following PCI remains inconclusive, especially in the case of an ACS, and further studies have to be conducted.

In conclusion, the optimal management of HBR patients who undergo PCI represent one of the most demanding challenges of contemporary interventional cardiology. The antithrombotic regimen, its duration, and the stents which should be implanted in HBR patients have to be planned thoroughly before the procedure. Our narrative review provides analytical algorithms to properly manage the HBR population and summarizes the current knowledge. Even though, in recent years, many clinical studies have been performed, more research need to be focused on HBR patients with concomitant high thrombotic burden, like those with an MI.

## Figures and Tables

**Table 1:** The ARC–HBR criteria. CKD: chronic kidney disease; eGFR; estimated glomerular filtration rate; ICH: intracranial hemorrhage.

Major Criteria	Minor Criteria
Anticipated use of long-term oral anticoagulation	Age ≥ 75 years
Severe or end-stage CKD (eGFR < 30 mL/min)	Moderate CKD (eGFR 30–59 mL/min)
Hemoglobin < 11 g/dL	Hemoglobin 11–12.9 g/dL for men and 11–11.9 g/dL for women
Spontaneous bleeding requiring hospitalization or transfusion in the past 6 months or at any time, if recurrent	Spontaneous bleeding requiring hospitalization or transfusion within the past 12 months not meeting the major criterion
Moderate or severe baseline thrombocytopenia (platelet count < 100 × 10^9^/L)	Long-term use of oral non-steroidal anti-inflammatory drugs or steroids
Chronic bleeding diathesis	Any ischemic stroke at any time not meeting the major criterion
Liver cirrhosis with portal hypertension	
Active malignancy (excluding non-melanoma skin cancer) within the past 12 months	
Previous spontaneous ICH (at any time)	
Previous traumatic ICH within the past 12 months	
Presence of a brain arteriovenous malformation	
Moderate or severe ischemic stroke within the past 6 months	
Non-deferrable major surgery on dual antiplatelet therapy	
Recent major surgery or major trauma within 30 days before percutaneous coronary intervention	

**Table 2:** Major clinical trials of HBR patients without an OAC indication undergoing PCI. ACS: acute coronary syndrome; BARC: bleeding academic research consortium; CCS: chronic coronary syndrome; CI: confidence interval; CRNM: clinically relevant non-major; CV: cardiovascular; CVD: cardiovascular death; DAPT: dual antiplatelet therapy; HBR: high bleeding risk; HR: hazard ratio; MACCE: major adverse cardiac and cerebrovascular event; MI: myocardial infarction; NACE: net adverse clinical event; OAC: oral anticoagulant; PCI: percutaneous coronary intervention; SAPT: single antiplatelet therapy; ST: stent thrombosis; TIMI: thrombolysis in myocardial infarction; TVR: target vessel revascularization. * High bleeding risk: clinical indication for treatment with oral anticoagulant (OAC) for at least 12 months, recent (<12 months) nonaccess site bleeding episode(s) that required medical attention (i.e., actionable bleeding), previous bleeding episode(s) that required hospitalization if the underlying cause had not been definitively treated (i.e., surgical removal of the bleeding source), age ≥75 years, systemic conditions associated with an increased bleeding risk (e.g., hematological disorders), including a history of current thrombocytopenia defined as a platelet count <100.00/mm^3^ (<100 × 10^9^/L) or any known coagulation disorder associated with increased bleeding risk, documented anemia, defined as repeated hemoglobin levels <11 g/dL or transfusion during the 4 weeks before inclusion, need for chronic treatment with steroids or non-steroidal anti-inflammatory drugs, diagnosed malignancy (other than skin) considered at high bleeding risk including gastrointestinal, genitourethral/renal and pulmonary, stroke at any time or transient ischemic attack in the previous 6 months, PRECISE-DAPT score ≥25.

Study	HBR Sample Size	ACS Patients	CCS Patients	Intervention Group	HBR Definition	Control Group	Duration	Primary Endpoint	Major Secondary Endpoints	Results
TWILIGHT-HBR (SUBGROUP ANALYSIS)	1064	661 (62.1%)	403 (37.9%)	Ticagrelor monotherapy after 3 months of DAPT with aspirin and ticagrelor	ARC–HBR criteria	Ticagrelor and aspirin	12 months	BARC type 2, 3 and 5 bleeding	Composite endpoint of all-cause death, MI or stroke, Major BARC 3 and 5 bleeding	Ticagrelor monotherapy reduced the incidence of the primary endpoint (*p* < 0.001) and major BARC 3 and 5 bleeding (*p* = 0.003). Composite endpoint of all-cause death, MI or stroke was similar between the 2 groups (*p* = 0.554).
TICO HBR (SUBGROUP ANALYSIS)	453	453 (100%)	0 (0%)	Ticagrelor monotherapy after 3 months of DAPT with aspirin and ticagrelor	ARC–HBR criteria	Ticagrelor and aspirin	12 months	NACE (composite of major bleeding and MACCE: all-cause death, MI, ST, stroke, TVR)	Major bleeding MACCEs	Ticagrelor monotherapy reduced NACE [HR (95% CI) = 0.30 (0.11–0.80)], and major bleeding [HR (95% CI) = 0.10 (0.01–0.81)]. MACCE was similar between the 2 groups [HR (95% CI) = 0.46 (0.15–1.47)].
STOPDAPT-2 HBR (SUBGROUP ANALYSIS)	1054	310 (29.4%)	744 (70.6%)	Clopidogrel monotherapy after 1-month DAPT (aspirin and clopidogrel)	ARC–HBR criteria	Aspirin and clopidogrel	12 months	Composite of CV (CVD, MI, definite ST, ischemic or hemorrhagic stroke) and bleeding (TIMI major/minor) outcomes	CV endpoint: (CVD, MI, definite ST, ischemic or hemorrhagic stroke) Bleeding endpoint (TIMI major/minor)	The primary endpoint (*p* = 0.06), and the major secondary cardiovascular endpoint (*p* = 0.43) were similar between the 2 groups. Major secondary bleeding endpoint was lower in the abbreviated DAPT group compared with the default strategy (*p* = 0.01).
MASTERDAPT (HBR TRIAL)	2913	1508 (51.8%)	1405 (48.2)	DAPT for 1 month and SAPT afterwards	One or more criteria for high bleeding risk *	DAPT for at least 6 months. After DAPT, aspirin monotherapy	335 days	NACE: a composite of all-cause death, MI, stroke, or major bleeding MACCE: a composite of all-cause death, MI, or stroke Major or CRNM bleeding	Individual components of the three primary outcomes	NACE (*p* = 0.91) and MACCE (*p* = 0.67) were similar between the 2 groups. Major or CRNM bleeding was lower in the abbreviated therapy group (*p* < 0.001).
STOPDAPT-3 HBR (SUBGROUP ANALYSIS)	3258	1803 (55.3%)	1455 (44.7%)	DAPT (aspirin with prasugrel 3.75 mg/day) for 1 month and aspirin afterwards up to 12 months	ARC–HBR criteria	Prasugrel monotherapy (3.75 mg/day) for 1 month and clopidogrel afterwards up to 12 months	1 month	Co-primary bleeding endpoint of bleeding BARC 3 and 5 Co-primary CV endpoint (composite of CVD, MI, definite ST, or ischemic stroke)	The major secondary endpoint was defined as a composite of the co-primary bleeding and CV endpoints representing net adverse clinical outcomes	The co-primary bleeding endpoint did not differ between the 2 groups both in the ACS (*p* = 0.59) and the non-ACS HBR patients (*p* = 0.84). The co-primary cardiovascular endpoint was numerically higher in the prasugrel monotherapy group only in ACS patients compared with the DAPT group (*p* = 0.07), while the 2 regimens did not differ in the non-ACS patients (*p* = 0.44).
TALOS-AMI HBR (SUBGROUP ANALYSIS)	589	589 (100%)	0 (0%)	After 1-month DAPT (aspirin and ticagrelor) de-escalation to aspirin and clopidogrel	Modified ARC–HBR criteria	Aspirin and ticagrelor	12 months	NACE (a composite of CVD, MI, stroke or bleeding BARC 2, 3 and 5)	Major bleeding BARC 3 or 5	NACE (*p* = 0.0053) and major bleeding BARC 3 or 5 (*p* = 0.0151) were lower in the de-escalation group.

**Table 3:** Major clinical trials of HBR patients with an OAC indication undergoing PCI. ACS: acute coronary syndrome; CCS: chronic coronary syndrome; CRNM: clinically relevant non-major; CVD: cardiovascular death; DAPT: dual antiplatelet therapy; DAT: dual antithrombotic therapy; HBR: high bleeding risk; MACCE: major adverse cardiac and cerebrovascular event; MACE: major adverse cardiovascular events; MI: myocardial infarction; NACE: net adverse clinical event; OAC: oral anticoagulant; PCI: percutaneous coronary intervention; SAPT: single antiplatelet therapy; ST: stent thrombosis; TAT: triple antithrombotic therapy; TIMI: thrombolysis in myocardial infarction; TVR: target vessel revascularization, VKA: vitamin K antagonist.

Study	HBR Sample Size	ACS Patients	CCS Patients	Intervention Group	Control Group	Duration	Primary Endpoint	Major Secondary Endpoints	Results
WOEST	563	155 (27.3%)	408 (72.7%)	OAC + clopidogrel	OAC + clopidogrel + aspirin	12 months	Any bleeding episode	Composite ischemic endpoint of death, MI, ST, TVR and stroke	Any bleeding was lower in the DAT group (*p* < 0.0001), and while major bleeding was similar between the 2 groups, the combined ischemic endpoint was also lower in the DAT group (*p* = 0.025).
ISAR TRIPLE	614	197 (32%)	417 (68%)	Clopidogrel for 6 weeks, aspirin and VKA	Clopidogrel for 6 months, aspirin and VKA	9 months	Composite endpoint of death, MI, definite ST, stroke, TIMI major bleeding	Composite ischemic endpoint (cardiac death, MI, definite ST, ischemic stroke) TIMI major bleeding	The primary cumulative endpoint was similar between the 2 groups (*p* = 0.63), as well as the secondary ischemic (*p* = 0.87) and bleeding endpoints (*p* = 0.44).
PIONEER AF-PCI	2124	1096 (52%)	1028 (48%)	-Rivaroxaban 15 mg + P2Y12 inhibitor (for 12 months, group 1) -Rivaroxaban 2.5 mg × 2 + DAPT (for 1, 6 or 12 months, group 2)	VKA + DAPT (for 1, 6 or 12 months, group 3)	12 months	TIMI major or minor bleeding	MACE (CVD, MI or stroke)	TIMI major or minor bleeding was lower in rivaroxaban groups (group 1: 16.8% vs. group 3: 26.7%, *p* < 0.001, group 2: 18% vs. group 3: 26.7%, *p* < 0.001), rates of CVD, MI, or stroke were similar in the 3 groups.
AUGUSTUS	4614	1714 (37.3%) treated with PCI, 1097 (23.9%) medically treated	1784 (38.8%)	(Two-by-two factorial design) P2Y12 inhibitor + apixaban or VKA + aspirin or placebo (for 6 months)	(Two-by-two factorial design) P2Y12 inhibitor + apixaban or VKA + aspirin or placebo (for 6 months)	6 months	Major or CRNM bleeding	Composite endpoint of death or hospitalization Composite endpoint of death or ischemic events (stroke, MI, ST, urgent revascularization)	The primary outcome occurred in 10.5% of the patients in apixaban group compared with 14.7% in the VKA group (*p* < 0.001 for both non-inferiority and superiority), and in 16.1% of the patients receiving aspirin compared with 9% of those receiving placebo (*p* < 0.001). Apixaban group had also lower rates of death or hospitalization compared with the VKA group (*p* = 0.002), and similar rates of ischemic events. Patients in the aspirin group had an incidence of death or hospitalization and ischemic events similar to the placebo group.
REDUAL PCI	2725	1375 (50.4%)	1350 (49.6%)	Dabigatran 110 mg × 2 + P2Y12 inhibitor -Dabigatran 150 mg × 2 + P2Y12 inhibitor	Warfarin + P2Y12 inhibitor + aspirin (1–3 months)	14 months (mean follow-up)	Major or CRNM bleeding	Composite efficacy endpoint of thromboembolic events (MI, stroke, systemic embolism), death, or unplanned revascularization	Major or CRNM bleeding was significantly lower in the 110 mg-DAT group compared with TAT. Major or CRNM bleeding was non-inferior in the 150 mg-DAT group compared with TAT. The composite efficacy endpoint was similar between the 3 groups.
ENTRUST AF-PCI	1506	777 (52%)	729 (48%)	Edoxaban + P2Y12 inhibitor (for 12 months)	VKA + P2Y12 inhibitor (for 12 months) + aspirin (for 1–12 months)	12 months	Major or CRNM bleeding	Composite endpoint of CVD, stroke, systemic embolization, MI, or definite ST	Major or CRNM bleeding complications occurred in 128 (17%) of 751 patients in the edoxaban group and in 152 (20%) of 755 patients in the VKA group (*p* = 0.001 for non-inferiority; *p* = 0.1154 for superiority), with similar ischemic events between the 2 groups.
MASTERDAPT	1666	703 (42%)	963 (58%)	1-month DAPT, SAPT for 5 additional months + OAC	DAPT for at least 3 months, SAPT thereafter till 12 months +OAC	335 days	NACE: a composite of all-cause death, MI, stroke, or major bleeding MACCE: a composite of all-cause death, MI, or stroke Major or CRNM bleeding	Individual components of the three primary outcomes	NACE (*p* = 0.26), MACCE (*p* = 0.53) and major or CRNM bleeding (*p* = 0.25) were similar between the abbreviated and the standard therapy.

**Table 4:** Major clinical trials of the stents which should be used in HBR patients undergoing PCI. BARC: bleeding academic research consortium; CD: cardiac death; CRNM: clinically relevant non-major; DAPT: dual antiplatelet therapy; HBR: high bleeding risk; MACCE: major adverse cardiac and cerebrovascular event; MI: myocardial infarction; NACE: net adverse clinical event; OAC: oral anticoagulant; PCI: percutaneous coronary intervention; SAPT: single antiplatelet therapy; ST: stent thrombosis; TLF: target lesion failure; TLR: target lesion revascularization; TVMI: target vessel myocardial infarction.

Study (Stent)	HBR Sample Size	Intervention Group	Control Group	Duration	Primary Endpoint	Major Secondary Endpoints	Results
MASTERDAPT (ULTIMASTER)	4579	Ultimaster stent with: DAPT for 1 month and SAPT afterwards (without OAC indication) 1-month DAPT, SAPT for 5 additional months + OAC (with OAC indication)	Ultimaster stent with: DAPT for at least 6 months. After DAPT, aspirin monotherapy (without OAC indication) DAPT for at least 3 months, SAPT thereafter till 12 months + OAC (with OAC indication)	335 days	NACE: a composite of all-cause death, MI, stroke, or major bleeding MACCE: a composite of all-cause death, MI, or stroke Major or CRNM bleeding	Individual components of the three primary outcomes	One-month DAPT was non-inferior compared with the standard regimen in the primary NACE (*p* < 0.001 for non-inferiority), and in the primary MACCE outcome (*p* = 0.001 for non-inferiority) The abbreviated-regimen group suffered fewer major or CRNM bleeding (*p* < 0.001 for superiority)
XIENCE SHORT DAPT PROGRAM (XIENCE)	3652	Xience stent with: 1-month DAPT and SAPT afterwards 3-month DAPT and SAPT afterwards	6-month DAPT historical control group 12-DAPT month historical control group	12 months	Composite endpoint of all-cause death or MI	Bleeding BARC types 2–5	The combined endpoint was similar between the 3-month DAPT group and the 12-month DAPT historical control group (5.4% vs. 5.4%, *p* for non-inferiority = 0.0063) as well as between the 1-month DAPT group and the 6-month DAPT historical control group (3.5% vs. 4.3%, *p* for non-inferiority = 0.0005). Major bleeding BARC 3–5 was lower in both the abbreviated DAPT groups compared with their historical controls
ONYX ONE (RESOLUTE ONYX)	1996	Resolute Onyx stent with one-month DAPT and SAPT afterwards	BioFreedom stent with one-month DAPT and SAPT afterwards	12 months	Safety composite outcome of CD, MI, or ST	Effectiveness outcome of TLF (CD, TVMI, TLR)	The Resolute Onyx stent was non-inferior to the BioFreedom stent in the composite primary endpoint (*p* = 0.01 for non-inferiority) and in the secondary endpoint of target lesion failure (*p* = 0.007 for non-inferiority)
EVOLVE SHORT DAPT (SYNERGY)	2009	Synergy stent with 3-month DAPT and aspirin afterwards	Historical control group	12 months after DAPT discontinuation	Two co-primary endpoints: Death or MI Definite/probable ST	Bleeding BARC (types 2, 3, 5)	The rate of MI or death was 5.6% compared with 5.7% of the prolonged DAPT historical control group (*p* for non-inferiority = 0.0016). The rate of definite/probable ST was lower than the prespecified performance goal (*p* = 0.0005)
BIOFLOW–DAPT (ORSIRO)	1948	Orsiro stent with one-month DAPT and SAPT afterwards	Resolute Onyx stent with one-month DAPT and SAPT afterwards	12 months DAPT	Composite outcome of CD, MI or probable or definite ST	Components of the primary outcome	The primary cumulative endpoint of CD, MI, or ST was similar between the 2 groups (*p* < 0.0001 for non-inferiority)
LEADERS FREE (BIOFREEDOM)	2466	BioFreedom stent with one-month DAPT and SAPT afterwards	Bare-metal stent with one-month DAPT and SAPT afterwards	390 days	Composite safety endpoint of CD, MI and ST Efficacy endpoint of clinically driven TLR	Bleeding BARC types 1–5	The BioFreedom stent was superior to Bare-metal stent regarding the composite safety endpoint (*p* < 0.001 for non-inferiority and *p* = 0.005 for superiority) and the efficacy endpoint (*p* < 0.001) Bleeding BARC types 1–5, 2–5 and 3–5 were similar between the 2 groups

## Data Availability

No new data were created or analyzed in this study. Data sharing is not applicable.

## Abbreviations

ACC/AHA	American college of cardiology/American heart association
ACS	Acute coronary syndrome
AF	Atrial fibrillation
APT	Antiplatelet therapy
ARC–HBR	Academic research consortium for high bleeding risk
AUGUSTUS	An open-label, 2 × 2 factorial, randomized controlled clinical trial to evaluate the safety of apixaban vs. vitamin k antagonist and aspirin vs. placebo
BARC	Bleeding academic research consortium
BMS	Bare-metal stent
CAD	Coronary artery disease
CCS	Chronic coronary syndrome
CD	Cardiac death
CI	Confidence interval
CKD	Chronic kidney disease
CREDO–Kyoto	Coronary revascularization demonstrating outcome study in Kyoto
CRNM	Clinically relevant non-major
CRUSADE	Can rapid risk stratification of unstable angina patients suppress adverse outcomes with early implementation of the ACC/AHA guidelines
CVD	Cardiovascular death
DAPT	Dual antiplatelet therapy
DAT	Dual antithrombotic therapy
DCB	Drug-coated ballon
DES	Drug-eluting stent
DM	Diabetes mellitus
DOAC	Direct oral anticoagulant
eGFR	Estimated glomerular filtration rate
ENTRUST–AF PCI	Edoxaban treatment vs. vitamin k antagonist in patients with atrial fibrillation undergoing percutaneous coronary intervention
EPIDAURUS	Escalated single-platelet inhibition for one month plus direct oral anticoagulation in patients with atrial fibrillation and acute coronary syndrome undergoing percutaneous coronary intervention
ESC	European society of cardiology
GLOBAL LEADERS	A clinical study comparing two forms of antiplatelet therapy after stent implantation
HBR	High bleeding risk
HOST–BR	Harmonizing optimal strategy for treatment of coronary artery diseases–dual antiplatelet therapy duration according to the bleeding risk
HOST–IDEA	Harmonizing optimal strategy for treatment of coronary artery stenosis coronary intervention with next-generation drug-eluting stent platforms and abbreviated dual antiplatelet therapy
HR	Hazard ratio
ICH	Intracranial hemorrhage
ISAR–TRIPLE	Triple therapy in patients on oral anticoagulation after drug-eluting stent implantation
LM	Left main
LoE	Level of evidence
MACCE	Major adverse cardiac and cerebrovascular events
MACE	Major adverse cardiovascular events
MASTERDAPT	Management of high-bleeding-risk patients post bioresorbable polymer coated stent implantation with an abbreviated versus standard dual antiplatelet therapy regimen
MI	Myocardial infarction
NACE	Net adverse clinical event
NSTEMI	Non–ST–segment elevation myocardial infarction
NSAIDS	Non-steroidal anti-inflammatory drugs
OAC	Oral anticoagulant
OMT	Optimal medical treatment
OPT–BIRISK	Optimal antiplatelet therapy for high bleeding and ischemic risk patients
PAD	Peripheral arterial disease
PARIS	Patterns of non-adherence to antiplatelet regimens in stented patients
PCI	Percutaneous coronary intervention
PIONEER AF–PCI	Prevention of embolic and neurological events in patients with atrial fibrillation undergoing percutaneous coronary intervention
PPI	Proton pump inhibitor
PRESICE DAPT	Predicting bleeding complications in patients undergoing stent implantation and subsequent dual antiplatelet therapy
PREMIUM	Prasugrel monotherapy following primary percutaneous coronary intervention for ST-elevation myocardial infarction
RCT	Randomized clinical trial
REDUAL PCI	Randomized evaluation of dual antithrombotic therapy with dabigatran vs. triple therapy with warfarin in patients with atrial fibrillation undergoing percutaneous coronary intervention
SAPT	Single antiplatelet therapy
ST	Stent thrombosis
STEMI	ST-segment-elevation myocardial infarction
STOPDAPT–2	Short and optimal duration of dual antiplatelet therapy after drug-eluting stent implantation–2
STOPDAPT–3	Short and optimal duration of dual antiplatelet therapy–3
T–PASS	Ticagrelor monotherapy in patients treated with new-generation drug-eluting stents for acute coronary syndrome
TALOS–AMI HBR	De-escalation from ticagrelor to clopidogrel in patients with acute myocardial infarction
TAT	Triple antithrombotic therapy
TICO	Ticagrelor monotherapy vs. ticagrelor with aspirin on major bleeding and cardiovascular events in patients with acute coronary syndrome
TIMI	Thrombolysis in myocardial infarction
TLF	Target lesion failure
TLR	Target lesion revascularization
TVMI	Target vessel myocardial infarction
TVR	Target vessel revascularization
TWILIGHT	Ticagrelor with or without aspirin in high-risk patients after percutaneous coronary intervention
TWILIGHT–ACS	Ticagrelor alone vs. ticagrelor plus aspirin following percutaneous coronary intervention in patients with non-ST-segment elevation acute coronary syndromes
TWILIGHT–HBR	Ticagrelor monotherapy in patients at high bleeding risk undergoing percutaneous coronary intervention
UA	Unstable angina
ULTIMATE–DAPT	Ultra-short versus standard dual antiplatelet therapy
VKA	Vitamin k antagonist
WOEST	What is the optimal antiplatelet and anticoagulant therapy in patients with oral anticoagulation and coronary stenting
ZES	Zotarolimus-eluting stents
ZEUS	Zotarolimus-eluting endeavor sprint stent in uncertain drug-eluting stent candidates
